# 180. Alterations to the Gut Microbiomes and Acquisition of Bacteria Resistance Elements among US International Travelers

**DOI:** 10.1093/ofid/ofab466.180

**Published:** 2021-12-04

**Authors:** Sushmita Sridhar, Colin Worby, Ryan Bronson, Sarah Turbett, Jason Harris, Edward T Ryan, Ashlee Earl, Regina C LaRocque

**Affiliations:** 1 Massachusetts General Hospital, Boston, MA; 2 Broad Institute of MIT and Harvard, Cambridge, Massachusetts

## Abstract

**Background:**

This study investigated the impact of international travel on the acquisition and carriage of antimicrobial resistance (AMR). We prospectively assessed U.S. international travelers for the acquisition of resistant *Enterobacterales *species and evaluated changes in travelers’ gut microbiomes.

**Methods:**

Metagenomic sequencing was performed on DNA extracted from pre- and post-travel stool samples of 273 U.S. international travelers. We used Kraken2 to assess microbial gut composition and analyzed antibiotic resistance gene (ARG) content using the Resistance Gene Identifier (RGI) and ResFinder, and read mapping to ARG databases. We assessed the change in gut profile and resistome associated with (i) all international travel; (ii) travel to specific geographic regions; and (iii) traveler’s diarrhea.

**Results:**

International travel resulted in a perturbation of the gut microbiome, which was greater in travelers receiving treatment for diarrhea during travel (*p* = 4E-5). There was an overall loss in microbial diversity following travel, regardless of health outcome (*p* = 0.011); this was most consistently observed in travelers to South East Asia (SEA) (loss of gut diversity in 81% of SEA travelers). 78% of all travelers had a higher relative abundance of *E. coli* after travel, including 85% of travelers who acquired AMR bacteria during travel. Travel to South Asia was also associated with a significantly greater increase of *E. coli* relative to other destinations (*p* = 0.04). Additionally, the relative abundance of *Pasteurellales* was higher in the pre-travel samples of those who subsequently acquired AMR bacteria (FDR = 0.08). Furthermore, there was a significant increase in ARG content among the post-travel samples, with regional differences in the magnitude of acquisition (**Figure 1**). 72% of all travelers had a greater resistance burden post-travel. SEA was associated with the greatest increase in resistome diversity, while South America was associated with the greatest increase in overall ARG content.

Resistance genes present in the gut microbiome.

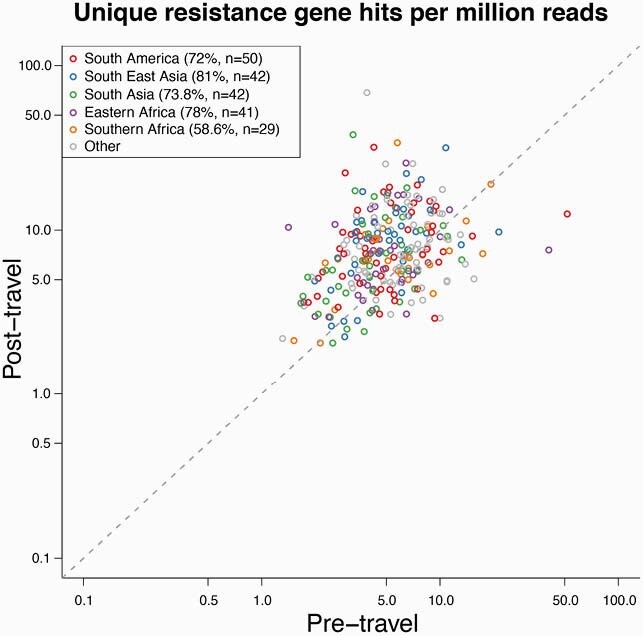

Genes mapping to the Comprehensive Antibiotic Resistance Database were measured pre- (x-axis) and post-travel (y-axis) to assess the acquisition of resistance genes in association with travel, distinguished by geographic region. Colors indicate geographic regions visited by travelers: South America (red), South East Asia (blue), South Asia (green), Eastern Africa (purple), Southern Africa (orange), Other (grey).

**Conclusion:**

International travel is associated with a perturbation in the gut microbial community, with the acquisition of AMR bacteria and genes, and an increase in the relative abundance of *E. coli*. These perturbations following travel may be important factors in the global spread of AMR.

**Disclosures:**

**All Authors**: No reported disclosures

